# Conversational artificial intelligence compared with orthodontic reference standards for orthodontic information: A systematic review of accuracy, readability, safety, and certainty of evidence

**DOI:** 10.1016/j.jobcr.2026.101552

**Published:** 2026-09-27

**Authors:** Karla Nogueira Matos, Giovanna Fontgalland Ferreira, Marcos de Faria Almeida, Leonardo Matos de Faria Almeida, Renato Araujo Prates, Hugo Henrique dos Santos Dantas Guimarães

**Affiliations:** a Department of Dentistry, University of São Paulo, Professor Lineu Prestes Avenue, 2227, São Paulo, SP, 05508-000, Brazil; b Department of Dentistry, Nove de Julho University, Diamantina Street, 302, 310, Vila Maria Baixa, São Paulo, SP, 02117-010, Brazil; c Department of Restorative Dentistry, São Leopoldo Mandic School of Dentistry, Dr José Rocha Junqueira Street, 13, Swift, Campinas, SP, 13045-755, Brazil; d Department of Restorative Dentistry, São Judas Tadeu University, Taquari Street, 546, Mooca, São Paulo, SP, 03166-000, Brazil; e Department of Restorative Dentistry, Federal University of Minas Gerais, Antônio Carlos Avenue, 6627, Pampulha, Belo Horizonte, MG, 31270-901, Brazil

**Keywords:** Artificial intelligence, Chatbots, Orthodontics, Patient education as topic, Decision support systems, Clinical, Health communication

## Abstract

**Background:**

Conversational artificial intelligence (AI) is increasingly used to obtain orthodontic information, but its reliability, completeness, readability, agreement with specialist judgment, and safety remain uncertain.

**Objective:**

To systematically evaluate AI chatbots for delivering orthodontic information and clinical guidance, focusing on accuracy/reliability, completeness, readability, specialist agreement, and misleading information.

**Methods:**

This systematic review followed PRISMA 2020 and Cochrane guidance. Embase, PubMed, Scopus, Web of Science, ClinicalTrials.gov, Open Science Framework, and Google Scholar were searched. Eligible studies assessed AI-generated orthodontic responses against specialists, reference answers, or structured assessment tools. Risk of bias was evaluated using the Joanna Briggs Institute checklist. Meta-analysis was not performed because of substantial heterogeneity in chatbot systems, prompts, comparators, scoring instruments, and outcome definitions. Certainty of evidence was assessed using GRADE.

**Results:**

Sixteen analytical cross-sectional studies were included. Chatbots generated coherent and accessible answers to routine orthodontic questions, especially foundational concepts and patient frequently asked questions. However, performance varied by model version, prompt design, and clinical complexity. Important limitations were identified in treatment planning, individualized recommendations, contextual risk framing, and information completeness. Some studies reported incomplete or false claims regarding clear aligners, airway effects, stability, and surgery. Readability was generally favorable but did not ensure clinical adequacy. Risk of bias ranged from low to moderate, and certainty of evidence was very low.

**Conclusions:**

AI chatbots may support routine orthodontic education under professional supervision, but current evidence does not support autonomous use. Standardized prompts, validated scoring tools, calibrated evaluators, and real-world outcome studies are needed.

## Highlights

• AI chatbots deliver orthodontic information with generally coherent content.
• Performance weakens in complex or individualized scenarios.
• Concordance with orthodontists is inconsistent.
• High readability does not ensure clinical adequacy.
• Evidence certainty remains very low.

## 1 Introduction

Artificial intelligence (AI) has rapidly transformed orthodontics, particularly through image-based diagnosis, automated measurements, treatment simulation, and decision-support systems 1, 2, 3. Recent systematic evidence has also shown that AI is increasingly being evaluated for diagnostic tasks in orthodontics, including the detection of orthodontic root resorption.^4^ Although diagnostic AI has received growing methodological attention, evidence regarding conversational AI systems used to generate orthodontic information for patients remains comparatively fragmented.

Conversational agents powered by large language models (LLMs) represent a distinct and increasingly visible application of AI in health communication. Unlike image-based or task-specific diagnostic systems, these platforms generate natural-language responses to user queries and may simulate interactive dialogue 5, 6, 7, 8, 9. Patients may therefore use chatbots to obtain explanations about appliances, treatment duration, risks, retention, clear aligners, orthognathic surgery, or treatment alternatives before consulting an orthodontist.^5^^,^^6^^,^^8^^,^^9^ This creates a clinically relevant interface between automated information and patient expectations.

Orthodontics is a complex context for chatbot-generated information because clinical decisions depend on individualized anatomical, functional, biomechanical, esthetic, and psychosocial factors, as well as legitimate variation in professional judgment and patient preferences. Thus, chatbot responses may seem fluent and plausible while omitting critical qualifiers, oversimplifying uncertainty, or presenting generalized information as patient-specific guidance.

The available primary studies suggest that AI chatbots can often provide coherent answers to routine orthodontic questions 5, 6, 7, 8, 9, 10, 11, 12, 13, 14. Nevertheless, important concerns remain regarding factual accuracy, completeness, readability, consistency across prompts, agreement with orthodontic specialists, and the risk of misleading or insufficiently contextualized information 12, 13, 14, 15, 16. These concerns are amplified by substantial methodological heterogeneity across studies, including differences in question sources, prompt construction, model versions, evaluator calibration, reference standards, and outcome instruments.

A recent systematic review evaluated patient trust in AI-generated orthodontic advice.^17^ However, the present review addresses a distinct and broader clinical-methodological question. Rather than focusing primarily on trust, this review evaluates accuracy, completeness, readability, specialist concordance, treatment-planning limitations, risk of misleading information, methodological heterogeneity, and certainty of evidence. Therefore, the present synthesis was designed to determine not only whether patients may trust AI-generated orthodontic advice, but whether chatbot-generated information is sufficiently accurate, complete, readable, and safe to support orthodontic communication under professional supervision.

Accordingly, this review synthesized evidence comparing AI chatbot-generated orthodontic information with specialist judgment, reference answers, or structured evaluation tools across five domains: accuracy/reliability, completeness, agreement with specialists, readability/accessibility, and risk of misleading or harmful information. By clarifying these domains and appraising certainty using the Grading of Recommendations Assessment, Development and Evaluation (GRADE) approach,^18^ this review aims to inform clinicians, educators, researchers, and policymakers regarding the safe and evidence-based integration of conversational AI into orthodontic communication.

## 2 Material and methods

### 2.1 Protocol and reporting standards

This systematic review was conducted according to the methodological guidance of the Cochrane Handbook^19^ for Systematic Reviews and reported in accordance with the Preferred Reporting Items for Systematic Reviews and Meta-Analyses (PRISMA) 2020 statement.^20^ The protocol, including eligibility criteria, outcomes, and planned synthesis strategy, was prospectively registered in the International Prospective Register of Systematic Reviews (PROSPERO)^21^ (CRD420261443844) before study selection.

### 2.2 Eligibility criteria

Eligibility criteria were defined according to a structured Population-Intervention-Comparator-Outcome framework.

The population (P) comprised orthodontic information contexts, including patient-facing questions, treatment explanations, appliance-related guidance, retention, clear aligner therapy, interceptive orthodontics, orthognathic surgery-related orthodontic information, treatment planning, and orthodontic education scenarios.

The intervention (I) was defined as responses generated by artificial intelligence-based conversational agents, including chatbots and large language models capable of producing natural-language orthodontic information.

Eligible comparators (C) included orthodontic specialists, expert panels, consensus-derived responses, textbooks, institutional materials, professional association information, predefined answer keys, or structured evaluative instruments.

Outcomes (O) of interest included accuracy, reliability, completeness, agreement with specialists or reference standards, readability, accessibility, and the presence of misleading or potentially harmful information.

Study design (S) comprised peer-reviewed original analytical, comparative, or evaluative studies, including cross-sectional studies, survey-based investigations, content analyses, validation or reliability studies, and other structured evaluations of chatbot-generated orthodontic information.

Original peer-reviewed analytical studies were eligible. Reviews, editorials, commentaries, conference abstracts, methodological papers without primary evaluative data, and studies not assessing chatbot-generated orthodontic information were excluded. A dedicated grey literature search of thesis repositories, dissertations, institutional reports, and unpublished documents was not performed. However, Google Scholar, Open Science Framework, and ClinicalTrials.gov were searched as complementary sources to identify potentially relevant records beyond traditional bibliographic databases. Eligibility was restricted to peer-reviewed analytical studies because the review aimed to synthesize evaluative evidence with sufficient methodological detail for critical appraisal and GRADE assessment.^18^

### 2.3 Information sources and search strategy

An updated electronic search was conducted in Embase, PubMed, Scopus, Web of Science, ClinicalTrials.gov, Open Science Framework, and Google Scholar. Search terms combined controlled vocabulary and free-text terms related to artificial intelligence, chatbots, large language models, and orthodontics. Search syntax was adapted to each database while preserving conceptual equivalence. The final search was conducted on May 9, 2026. The complete PICOS-based search strategies, including study-design/evaluation terms, and the number of records retrieved per source are provided in Supplementary Table S1.

### 2.4 Study selection

All records were imported into Rayyan,^22^ and duplicates were identified and removed before screening. Two reviewers independently (G.F.F. and M.F.A.) screened titles and abstracts. Potentially eligible reports were retrieved in full and assessed against the eligibility criteria. Disagreements were resolved through discussion, with arbitration by a third reviewer (K.N.M.) when necessary.

Reasons for exclusion at the full-text stage were recorded and grouped into predefined methodological categories, including lack of orthodontic AI-chatbot evaluation, absence of structured outcome assessment, editorial or narrative format, non-patient-facing or non-clinical context, overlapping datasets, and insufficient methodological detail. The study selection process is shown in Fig. 1.

**Fig. 1:**
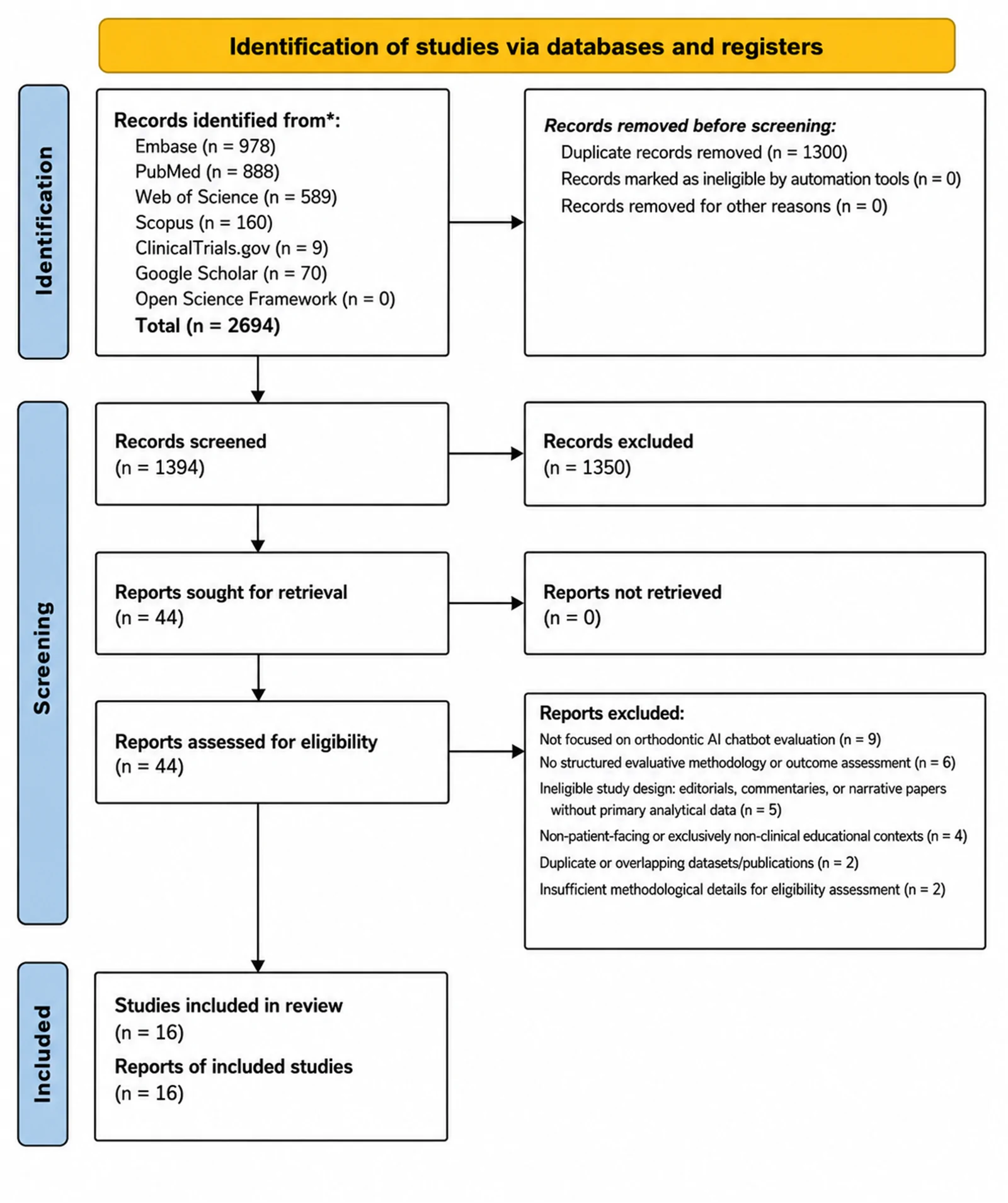
PRISMA 2020 flow diagram of study selection Flow diagram showing database identification, screening, eligibility assessment, exclusions with reasons, and included studies.

### 2.5 Data extraction

A standardized extraction form was used to collect bibliographic data, study design, orthodontic context, chatbot system, model version, access date, prompt source, question type, comparator, evaluator profile, assessment instruments, statistical methods, and main findings.

Special attention was given to variables highlighted by the reviewers as potential sources of heterogeneity, including prompt standardization, session resetting, evaluator calibration, blinding, comparator type, and whether questions reflected patient-facing information, educational testing, or clinical decision-making contexts.

Core characteristics of the included studies are summarized in Table 1. Technical details of the AI systems are presented in Supplementary Table S2, and prompt methodology, question sources, and thematic coverage are described in Supplementary Table S3.

**Table 1:** Characteristics of the studies included in the systematic review evaluating artificial intelligence-based conversational agents in orthodontics.

Study	Orthodontic context and sample	AI chatbot(s)/version	Comparator/evaluators	Evaluation outcomes/main focus
**Abu Arqub et al., 2024**^12^	Clear aligner treatment; 111 questions	ChatGPT; version NR	5 orthodontists/CAT experts; evidence-based rubric	Accuracy of AI-generated CAT claims; 4-point accuracy scale
**Alkhamees, 2024**^9^	Impacted canines, interceptive orthodontics, orthognathic surgery; 60 Q/A pairs	ChatGPT-4	5 experienced orthodontists	Accuracy and comprehensiveness; 5-point Likert
**Arslan et al., 2024**^5^	General orthodontic FAQs; 24 questions	ChatGPT; Google Bard	1 orthodontist + 1 resident; model comparison	Accuracy/quality; modified Likert, ICC, word/reference counts
**Daraqel et al., 2024**^6^	General orthodontic questions; 100 questions in 10 domains	ChatGPT-3.5; Google Bard; accessed July 16, 2023	5 blinded orthodontic assessors	Accuracy, completeness, response time/length; AOI, VAS, ICC
**Dursun and Bilici Gecer, 2024**^14^	Clear aligner patient FAQs; 20 questions	ChatGPT-3.5; ChatGPT-4; Gemini; Copilot	2 orthodontist evaluators	Accuracy, reliability, quality, readability; Likert, modified DISCERN, GQS, FRES
**Hatia et al., 2024**^16^	Interceptive orthodontics; 21 open questions + 7 clinical scenarios	ChatGPT-4; April 21, 2023	10 specialized orthodontists from postgraduate schools	Accuracy/completeness of answers and clinical scenarios; Likert scales
**Kılınç & Mansız, 2024**^13^	General and treatment-related FAQs; 34 questions	ChatGPT March 23 and May 24, 2023 versions	Version comparison; 2 orthodontists	Reliability and readability over time; DISCERN, Flesch-Kincaid, ICC
**Kurt Demirsoy et al., 2024**^10^	Clear aligners, lingual orthodontics, esthetic braces, TMD; 4 prompts	ChatGPT-4	35 orthodontists, 35 dental students, 35 patients	Reliability and quality; DISCERN and GQS
**Makrygiannakis et al., 2024**^15^	Clinically relevant orthodontic questions; 10 questions	ChatGPT-3.5; ChatGPT-4; Google Bard; Microsoft Bing	2 specialist orthodontists; evidence-based answers	Comprehensiveness, scientific accuracy, clarity, relevance; 0-10 rubric
**Metin & Goymen, 2025**^7^	General orthodontic knowledge; 40 true/false questions	ChatGPT-4; ChatGPT-4o; Copilot; Gemini 1.5 Pro; Claude 3.5 Sonnet	30 students, 30 dentists, 30 orthodontists; answer key	Accuracy and response consistency; percentage accuracy, Cronbach's alpha
**Naureen & Kiani, 2024**^23^	MARPE, clear aligners, CBCT; 90 questions	ChatGPT-4; Google Bard	2 independent raters; standard answer key	Knowledge accuracy; 5-point scoring, ICC, paired tests
**Salmanpour et al., 2025**^8^	AAO patient-facing FAQs; 15 questions	ChatGPT-4.0; Microsoft Copilot; Oct 5-25, 2024	Expert answers; 52 orthodontists + 102 patients	Adequacy of chatbot vs expert answers; 10-point Likert, Cronbach's alpha, Flesch-Kincaid
**Tanaka et al., 2025**^24^	Orthodontic treatment planning; 1 clinical case	ChatGPT-3.5; Bing Copilot; Google Bard/Gemini; Feb 2, 2024	Expert orthodontist plan; 34 orthodontists	Agreement with AI vs human treatment plans; Likert, VAS, ANOVA
**Vassis et al., 2024**^25^	Pretreatment side-effect education; 28 prompts + 46 patient perceptions	ChatGPT-3.5; ChatGPT-4; Oct 2023 version	3 experts + orthodontic patients; standard university text	Reliability, content validity, patient preference; Likert scales
**Yurdakurban et al., 2024**^26^	Orthognathic surgery patient information; 60 questions	ChatGPT-4; OpenEvidence; MediSearch	Researcher-developed guideline-based questions	Quality, reliability, readability, similarity; EQIP, DISCERN-adapted, GQS, SMOG
**Zhou et al., 2025**^11^	Orthodontic patient FAQs; 30 Chinese-language questions	ChatGPT-4; accessed Sept 16, 2023	2 orthodontists; 4 senior orthodontist raters	Ranking of AI vs orthodontists; Friedman, Nemenyi, Kendall W, Spearman

Abbreviations: AAO, American Association of Orthodontists; AI, artificial intelligence; ANOVA, analysis of variance; AOI, Accuracy of Information; CA, clear aligner; CAT, clear aligner treatment; CBCT, cone-beam computed tomography; DISCERN, consumer health-information assessment tool; EB, esthetic braces; EQIP, Ensuring Quality Information for Patients; FAQ, frequently asked question; FRES, Flesch Reading Ease Score; GPT, Generative Pre-trained Transformer; GQS, Global Quality Scale; ICC, intraclass correlation coefficient; IQR, interquartile range; LLM, large language model; LO, lingual orthodontics; MARPE, mini-implant-assisted rapid palatal expansion; NR, not reported; Q/A, questions and answers; SD, standard deviation; SMOG, Simple Measure of Gobbledygook; TMD, temporomandibular disorders; VAS, visual analog scale.

### 2.6 Outcome domains

The synthesis was organized around five prespecified domains: accuracy or reliability, completeness of information, agreement with specialists or reference standards, readability or accessibility, and risk of misleading or harmful information.

Because orthodontic decision-making may vary according to clinical philosophy, esthetic priorities, institutional training, and patient-specific factors, agreement with specialists was not treated as an absolute measure of correctness. Instead, it was interpreted as concordance with the specific reference framework used in each study.

### 2.7 Risk of bias assessment

Risk of bias was assessed using the Joanna Briggs Institute (JBI) Critical Appraisal Checklist for Analytical Cross-Sectional Studies. This tool was selected because all included studies used cross-sectional comparative or evaluative designs in which chatbot-generated responses were assessed at a specific time point against professional, institutional, or structured reference standards.

Two reviewers independently (M.F.A. and L.M.F.A.) assessed each study. Judgments were made qualitatively rather than by numerical scoring. Particular attention was paid to inclusion criteria, description of study setting or question source, validity of exposure and outcome measurement, evaluator calibration, blinding, confounding or contextual influences, and appropriateness of statistical analysis.

Domain-level judgments and overall risk-of-bias ratings are reported in Table 2.

**Table 2:** Risk of bias assessment of included studies using the Joanna Briggs Institute (JBI) Critical Appraisal Checklist for Analytical Cross-Sectional Studies.

Study	Q1	Q2	Q3	Q4	Q5	Q6	Q7	Q8	Overall risk of bias
Arslan et al., 2024^5^	**Y**	**Y**	**Y**	**P**	**P**	**N**	**P**	**Y**	**Moderate**
Daraqel et al., 2024^6^	**Y**	**Y**	**Y**	**Y**	**Y**	**Y**	**Y**	**Y**	**Low**
Dursun et al., 2024^14^	**Y**	**Y**	**Y**	**Y**	**P**	**N**	**Y**	**Y**	**Moderate**
Kılınç and Mansız, 2024^13^	**Y**	**Y**	**Y**	**P**	**P**	**N**	**Y**	**Y**	**Moderate**
Makrygiannakis et al., 2024^15^	**Y**	**Y**	**Y**	**Y**	**Y**	**P**	**Y**	**Y**	**Low**
Abu Arqub et al., 2024^12^	**Y**	**Y**	**Y**	**Y**	**P**	**N**	**Y**	**P**	**Moderate**
Alkhamees, 2024^9^	**Y**	**Y**	**P**	**P**	**P**	**N**	**P**	**Y**	**Moderate**
Hatia et al., 2024^16^	**Y**	**Y**	**Y**	**P**	**P**	**N**	**P**	**Y**	**Moderate**
Kurt Demirsoy et al., 2024^10^	**Y**	**Y**	**Y**	**P**	**P**	**N**	**Y**	**Y**	**Moderate**
Naureen and Kiani, 2024^23^	**Y**	**Y**	**Y**	**Y**	**Y**	**P**	**Y**	**Y**	**Low**
Vassis et al., 2024^25^	**Y**	**Y**	**P**	**Y**	**P**	**P**	**Y**	**Y**	**Moderate**
Yurdakurban et al., 2024^26^	**Y**	**Y**	**Y**	**Y**	**Y**	**P**	**Y**	**Y**	**Low**
Metin and Goymen, 2025^7^	**Y**	**Y**	**Y**	**Y**	**Y**	**P**	**Y**	**Y**	**Low**
Salmanpour et al., 2025^8^	**Y**	**Y**	**Y**	**P**	**P**	**N**	**P**	**Y**	**Moderate**
Tanaka et al., 2025^24^	**Y**	**Y**	**Y**	**P**	**P**	**N**	**P**	**Y**	**Moderate**
Zhou et al., 2025^11^	**Y**	**Y**	**Y**	**Y**	**Y**	**P**	**Y**	**Y**	**Low**

Legend: Y = yes/criterion adequately met; P = partial, unclear, or incompletely reported; N = no/not reported or insufficiently addressed. Q1, clearly defined inclusion criteria; Q2, detailed description of study subjects/settings or question/case source; Q3, valid and reliable measurement of exposure/intervention (AI model, access, prompting procedure); Q4, objective and standardized criteria for outcome assessment; Q5, identification of potential confounding or contextual factors; Q6, strategies to address confounding/contextual influences; Q7, valid and reliable outcome measurement; Q8, appropriate statistical analysis. Overall judgments were qualitative and did not rely on numerical scoring.

### 2.8 Data synthesis

Quantitative meta-analysis was not performed because of substantial clinical, methodological, and statistical heterogeneity across the included studies. Differences were identified in chatbot architectures, model versions, access dates, prompt-engineering strategies, question sources, comparator definitions, evaluator backgrounds, scoring instruments, and outcome operationalization. Because these studies did not estimate a common effect using comparable metrics or sufficiently homogeneous designs, pooled quantitative synthesis was considered inappropriate and potentially misleading. Therefore, findings were synthesized narratively according to outcome domains. This approach allowed integration of heterogeneous evidence while preserving distinctions between patient-facing information, educational knowledge testing, specialist agreement, treatment-planning performance, readability, and safety-related concerns.

Detailed comparative findings are provided in Supplementary Table S4. Additional exploratory domains, including patient preference, treatment-planning applicability, and perceived clinical usefulness, were visually synthesized in Fig. 2 to illustrate broader patterns of chatbot performance across studies.

**Fig. 2:**
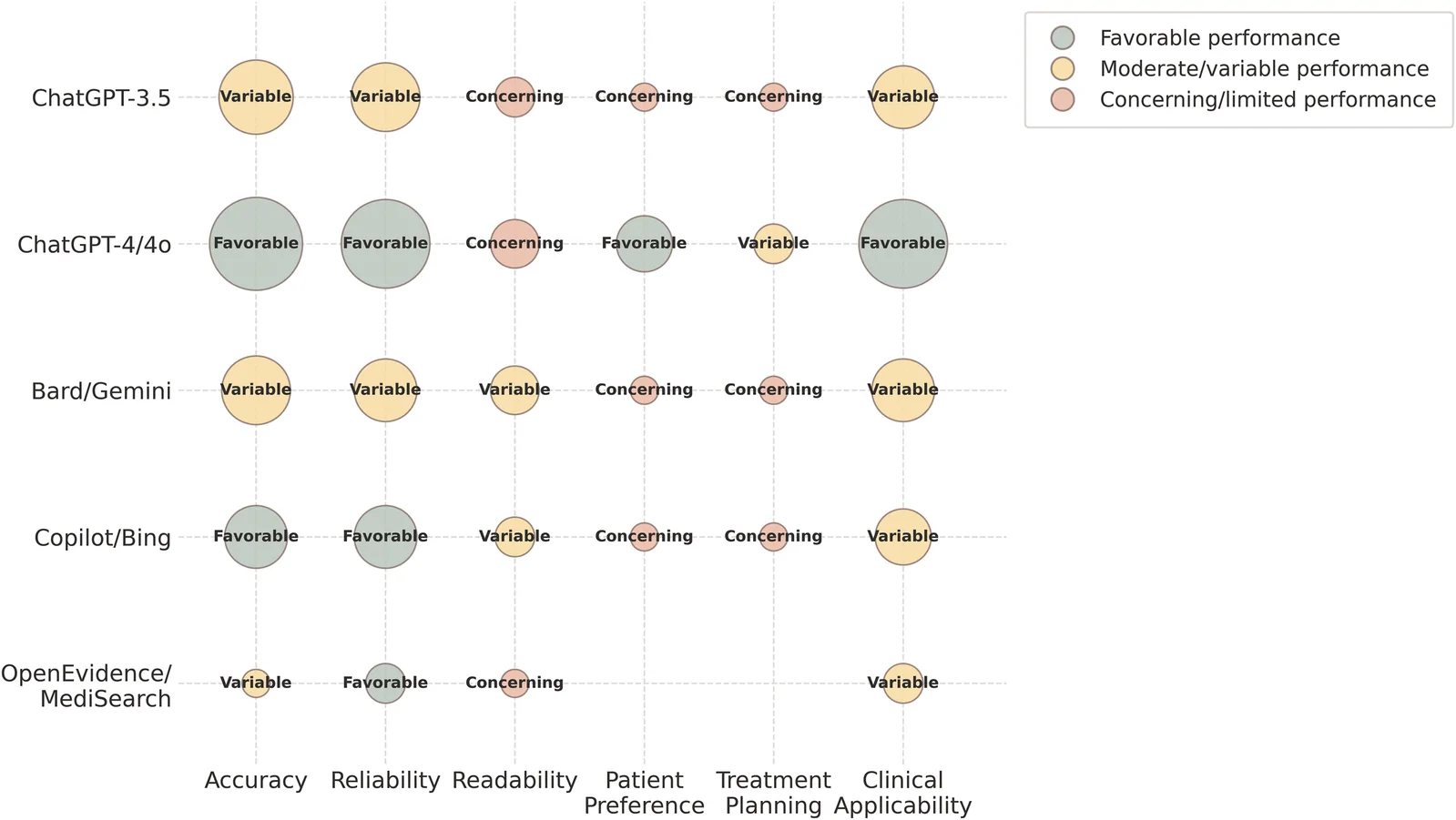
Evidence landscape of AI chatbot performance in orthodontics Visual synthesis of chatbot performance across orthodontic outcome domains. Bubble size reflects the relative amount of contributing evidence and does not represent pooled quantitative estimates.

A clinically oriented supervision framework was also developed from the narrative synthesis to translate these findings into proposed levels of professional oversight for orthodontic communication (Table 3 and Fig. 3).

**Table 3:** Proposed clinical supervision framework for conversational AI use in orthodontic information.

Clinical use category	Examples of chatbot use	Evidence pattern across included studies	Main clinical concern	Recommended supervision level
Routine orthodontic education and general FAQs	Definitions of orthodontics; basic treatment process; appliance care; oral hygiene; diet; treatment duration; retention basics; frequently asked patient questions.	Several FAQ-based studies reported coherent, generally acceptable, or high-scoring responses for routine questions. Examples include Daraqel et al. for 100 general orthodontic questions, Arslan et al. for 24 common questions, and Zhou et al. for Chinese-language patient FAQs.	General responses may be fluent but still omit patient-specific qualifiers, alternative options, or limitations.	Adjunctive use is reasonable after orthodontist review or when embedded in clinician-curated patient education. Responses should be labelled as general information, not individualized advice.
Patient information requiring contextual qualification	Clear aligner FAQs; side effects of orthodontic treatment; retention and relapse; pain management; orthognathic-surgery recovery; appliance choice.	Evidence was mixed. Dursun and Bilici Gecer found generally accurate clear-aligner responses but variable reliability, quality, and readability. Vassis et al. found that patients often preferred AI-generated education, whereas experts judged the content insufficient for standalone education. Yurdakurban et al. reported good quality/reliability for orthognathic-surgery information but difficult readability.	Risk of incomplete counselling, difficult readability, underemphasis of warnings, and overgeneralized recommendations.	Use only after professional verification. Add disclaimers, explain uncertainty, and align outputs with institutional patient information and readability standards.
Evidence-sensitive or claim-sensitive topics	Claims about clear aligner effectiveness; airway effects; sleep apnea; root control; treatment stability; surgery need; TMD-related statements; comparisons between appliances.	Abu Arqub et al. found that only 58% of ChatGPT-generated clear-aligner statements were objectively true and 15% were false, including claims related to surgery need, airway function, root parallelism, sleep apnea, and stability. Makrygiannakis et al. also reported that LLM answers occasionally lacked comprehensiveness, scientific accuracy, clarity, or relevance.	Misleading or unsupported statements may affect expectations, consent, and treatment decisions even when the language appears confident.	Do not release directly to patients without fact-checking. Orthodontist review against current evidence is required before use.
Educational or knowledge support for trainees and clinicians	True/false knowledge testing; textbook-based orthodontic concepts; MARPE, CBCT, clear aligner knowledge; evidence retrieval for clinician-oriented questions.	Metin and Goymen found that chatbots generally outperformed students and dentists but remained below orthodontists, with important consistency differences among models. Naureen and Kiani found ChatGPT-4 more accurate than Google Bard for recent orthodontic modalities. These studies support educational adjunct use but not unverified authority.	Inconsistent outputs and model-dependent performance can create false confidence in educational or clinical learning contexts.	Use as a supplementary educational tool with answer keys, instructor verification, and explicit discussion of model limitations.
Clinical case interpretation and treatment planning	Diagnosis; individualized treatment recommendations; extraction or non-extraction decisions; impacted tooth management; mixed-dentition planning; surgical and airway-related planning.	Tanaka et al. showed significantly greater orthodontist agreement with an expert orthodontist treatment plan than with AI-generated plans. Hatia et al. reported high but incomplete accuracy/completeness for interceptive orthodontic clinical questions and scenarios. Alkhamees reported generally good ChatGPT-4 responses, but with variability and low inter-rater agreement.	Individualized decisions require examination, records, risk assessment, patient preferences, and specialist judgment; apparent agreement with one reference does not prove correctness.	Not appropriate for autonomous use. Chatbots may support discussion or drafting, but diagnosis and treatment planning must remain specialist-led.
Implementation safeguards across all categories	Model/version documentation; access date; standardized prompts; session resetting; evaluator calibration; readability checking; evidence verification; disclosure of limitations.	Across studies, heterogeneity in prompts, models, access dates, comparators, and outcome tools limited synthesis. GRADE certainty remained very low for all outcomes.	Model drift, prompt sensitivity, lack of reproducibility, and non-standardized outcome assessment limit confidence in direct clinical transfer.	Institutional governance is recommended: use predefined prompts, audit outputs periodically, document model versions, and ensure patient-facing outputs direct users to orthodontic consultation.

Abbreviations: AI, artificial intelligence; CBCT, cone-beam computed tomography; FAQ, frequently asked question; GRADE, Grading of Recommendations Assessment, Development and Evaluation; LLM, large language model; MARPE, mini-implant-assisted rapid palatal expansion; TMD, temporomandibular disorders. The framework synthesizes reported evidence patterns and does not replace clinical judgment or locally validated patient-information protocols.

**Fig. 3:**
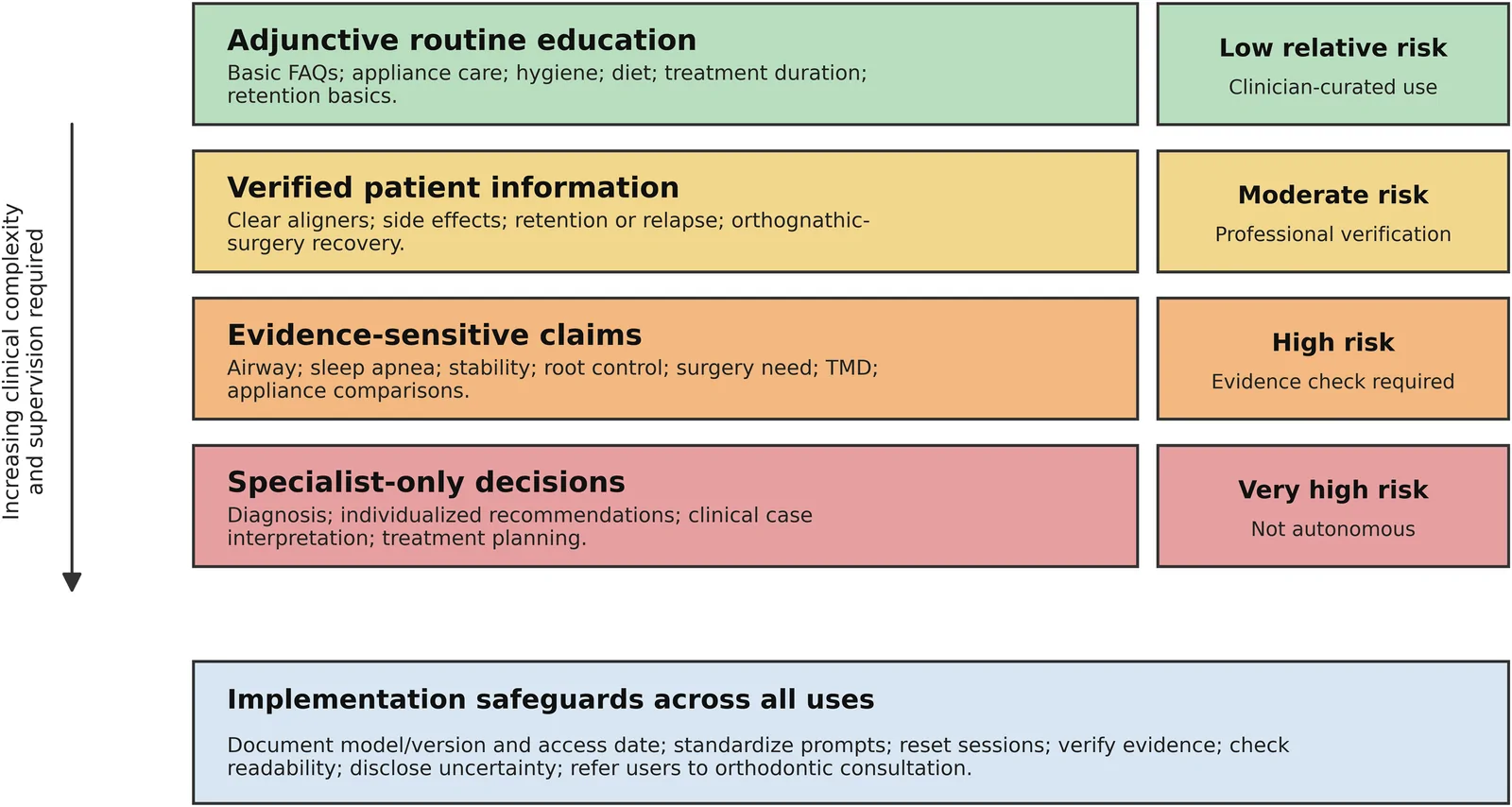
Clinical supervision framework for conversational artificial intelligence in orthodontic communication. Framework summarizing proposed levels of clinical supervision for conversational AI use in orthodontic communication. FAQ, frequently asked question; TMD, temporomandibular disorders.

### 2.9 Certainty of evidence

The certainty of evidence was assessed using the GRADE approach.^18^ Because all included studies were observational and cross-sectional in design, certainty initially started at low. Downgrading considered risk of bias, inconsistency, indirectness, imprecision, and other concerns.

Risk-of-bias judgments were informed by the JBI assessment. Inconsistency reflected variability in study conclusions and chatbot performance across domains. Indirectness was considered in relation to the applicability of simulated prompts and curated question banks to real-world orthodontic communication. Imprecision reflected small datasets, limited numbers of evaluators, absence of pooled estimates, and lack of standardized thresholds.

The certainty of evidence was summarized across five outcomes: accuracy/reliability, completeness, agreement with specialists, readability/accessibility, and risk of misleading or harmful information. The final GRADE Summary of Findings table is presented in Supplementary Material 2.

## 3 Results

### 3.1 Study selection

The search strategy identified 2694 records across electronic databases and registers, including Embase (n = 978), PubMed (n = 888), Web of Science (n = 589), Scopus (n = 160), ClinicalTrials.gov (n = 9), Google Scholar (n = 70), and the Open Science Framework (n = 0). After the removal of 1300 duplicate records, 1394 titles and abstracts were screened. Of these, 1350 records were excluded during initial screening. Forty-four reports were sought for retrieval, and all full texts were successfully obtained. Following the eligibility assessment, 28 reports were excluded for predefined methodological or conceptual reasons, resulting in 16 studies included in the final qualitative synthesis (Fig. 1).

The most common reasons for exclusion at full-text evaluation were studies not focused on orthodontic AI-chatbot evaluation (n = 9), absence of structured evaluative methodology or outcome assessment (n = 6), editorials or narrative papers without primary analytical data (n = 5), non-patient-facing or exclusively non-clinical educational contexts (n = 4), overlapping datasets (n = 2), and insufficient methodological detail (n = 2) (Fig. 1).

### 3.2 Study characteristics

The included investigations were published between 2024 and 2025, reflecting the rapid expansion of conversational artificial intelligence research in orthodontics. All studies employed analytical cross-sectional comparative designs evaluating chatbot-generated responses against orthodontic specialists, expert-derived reference standards, institutional materials, validated assessment tools, or combinations thereof. Core study characteristics are summarized in Table 1.

Substantial heterogeneity was identified regarding evaluated chatbot systems, prompt engineering strategies, question datasets, comparator definitions, and outcome frameworks. Investigated AI systems included ChatGPT-3.5, ChatGPT-4/4o, Google Bard/Gemini, Microsoft Copilot/Bing, Claude 3.5 Sonnet, OpenEvidence, and MediSearch. Access conditions also varied considerably, including free and paid versions, publicly available interfaces, and temporally distinct model releases (Supplementary Table S2).

Dataset construction differed markedly across studies. Some investigations used orthodontist-generated consensus question banks or evidence-based reference answers, whereas others extracted patient-oriented queries from Google searches, professional association frequently asked questions (FAQs), textbooks, or institutional educational materials. Question volumes ranged from a single complex clinical treatment-planning case to 111 open-ended prompts involving clear aligner therapy claims (Supplementary Table S3).

Importantly, not all included studies evaluated identical informational contexts. While most investigations simulated patient-facing orthodontic information-seeking scenarios, several studies explored educational, treatment-planning, or knowledge-testing environments involving residents, dental students, orthodontists, or mixed evaluator groups. Consequently, the synthesis focused on the comparative informational performance of AI-generated orthodontic content rather than exclusively on direct patient simulation models.

Considerable variability was also observed in prompt methodology. Some studies standardized prompts using predefined questions and independent sessions with history resetting, whereas others relied on self-formulated prompts, single-session interactions, or researcher-entered questions without explicit session-control protocols. These methodological differences likely influenced response consistency and contributed substantially to between-study heterogeneity (Supplementary Table S3).

### 3.3 Risk of bias within studies

Risk of bias assessments using the Joanna Briggs Institute Critical Appraisal Checklist are summarized in Table 2. Although several studies demonstrated acceptable methodological rigor, recurrent concerns regarding evaluator calibration, blinding, and standardization resulted in overall serious risk-of-bias considerations within the GRADE framework.^18^

Studies judged as lower risk more frequently reported clearly defined inclusion criteria, standardized prompting procedures, blinded evaluation strategies, and calibrated scoring frameworks. Investigations such as Daraqel et al.,^6^ Naureen et al.,^23^ Yurdakurban et al.,^26^ Metin and Goymen,^7^ and Zhou et al.,^11^ demonstrated stronger methodological transparency and more robust evaluator reliability procedures.

Conversely, moderate concerns frequently emerged from insufficient reporting regarding evaluator masking, confounding management, and calibration procedures. Several investigations relied on subjective expert judgment without formal validation or pilot calibration of assessment tools. In multiple studies, it remained unclear whether evaluators were blinded to chatbot identity, study objectives, or comparator origin, potentially introducing expectation bias into comparative ratings.

An additional source of heterogeneity involved comparator definitions. Some studies used individual orthodontists as reference standards, whereas others relied on consensus-derived answers, institutional educational materials, textbooks, or structured evidence-based frameworks. These comparator models are not conceptually equivalent and may partially explain differences observed in “agreement with specialists” outcomes across studies.

Evaluator calibration also varied considerably. Studies incorporating inter-rater reliability analyses, pilot scoring sessions, or standardized consensus procedures generally produced more reproducible assessments than studies lacking formal calibration methods. This distinction was considered important when interpreting the stability and credibility of comparative findings.

Despite these limitations, no study demonstrated a critical methodological failure incompatible with inclusion. Most investigations maintained reasonable alignment between objectives, study design, and analytical approaches, supporting their inclusion within an exploratory evidence synthesis framework.

### 3.4 Results of individual studies and synthesis of findings

Because of substantial heterogeneity in chatbot architectures, comparator frameworks, prompt methodologies, and scoring systems, quantitative pooling was not considered methodologically appropriate. Findings were therefore synthesized narratively according to prespecified outcome domains. Detailed comparative results are presented in Supplementary Table S4, whereas the global evidence landscape is illustrated in Fig. 2.

Across the included investigations, AI-based chatbots generally produced coherent and clinically plausible responses to routine orthodontic questions, particularly those involving foundational concepts, appliance explanations, oral hygiene instructions, treatment duration, or frequently asked patient concerns. However, performance became more variable when questions required individualized judgment, contextual interpretation, or treatment-planning reasoning.

### 3.5 Accuracy and reliability

Several studies reported favorable accuracy performance for ChatGPT-4 and related large language models when answering common orthodontic questions. Daraqel et al.^6^ observed high accuracy and completeness for both ChatGPT and Bard, although ChatGPT achieved significantly higher median accuracy scores. Similarly, Arslan et al.^5^ reported slightly superior performance for ChatGPT compared with Bard in orthodontic FAQ responses.

Nevertheless, clinically meaningful limitations persisted. Abu Arqub et al.^12^ demonstrated that only 58% of ChatGPT-generated statements regarding clear aligners were objectively correct, whereas 15% were considered false. False or potentially misleading claims involved surgery indications, airway effects, root control, stability, and sleep apnea.

Studies evaluating consistency across repeated sessions also identified variability in response reproducibility. Metin and Goymen^7^ observed differences in consistency and accuracy among ChatGPT-4, ChatGPT-4o, Gemini, Copilot, and Claude models, reinforcing that reliability remains dependent on model architecture and prompting context.

### 3.6 Completeness of information

Although many chatbot responses addressed the primary question adequately, omissions of clinically relevant contextual details were frequent. Several studies noted insufficient discussion of treatment limitations, individualized contraindications, risk modifiers, or alternative therapeutic options.

These omissions became particularly evident in studies involving orthognathic surgery, interceptive orthodontics, and clear-aligner therapy. While outputs were often linguistically fluent and superficially comprehensive, specialist evaluators frequently identified incomplete framing of risks, inadequate qualification of recommendations, or insufficient contextualization for individualized decision-making.

### 3.7 Agreement with specialists and treatment-planning performance

Agreement with orthodontists was inconsistent across studies and strongly dependent on question complexity. The lowest agreement levels were observed in treatment-planning scenarios and case-based reasoning.

Tanaka et al.^24^ demonstrated that AI-generated orthodontic treatment plans differed substantially from expert orthodontist recommendations when managing a mixed-dentition clinical case involving impacted incisors and transverse discrepancies. Similarly, studies comparing AI outputs with specialist-generated responses frequently found that orthodontists achieved superior ratings for adequacy, nuance, and contextual appropriateness.

Importantly, disagreement between specialists themselves represents an intrinsic challenge in orthodontics because therapeutic philosophies, esthetic preferences, and clinical priorities may vary across practitioners and institutions. Consequently, “agreement with specialists” should not be interpreted as an absolute binary construct, but rather as concordance with the reference framework adopted within each individual study.

### 3.8 Readability and accessibility

Readability was one of the most consistently favorable chatbot characteristics. Several investigations reported that AI-generated responses were accessible, easy to understand, and often written in patient-friendly language. In some studies, readability scores were comparable or superior to specialist-generated educational materials.

However, increased readability did not necessarily correspond to greater clinical adequacy. Kılınç and Mansız^13^ demonstrated that improvements in informational depth could reduce reading-ease scores, suggesting a trade-off between simplicity and technical completeness. Accordingly, readability should be interpreted as an independent communicative construct rather than a surrogate marker of clinical reliability.

### 3.9 Risk of misleading or harmful information

Overtly dangerous recommendations were uncommon across the included literature. However, several investigations highlighted the possibility of indirect harm through omission of warnings, oversimplification, overgeneralization, or confident phrasing lacking appropriate clinical qualification.

Examples included generalized recommendations regarding clear aligners, unsupported assumptions about airway improvement, simplified orthognathic surgery expectations, and treatment suggestions lacking individualized diagnostic assessment. These findings reinforce that conversational fluency alone should not be interpreted as evidence of clinical validity or safety.

Overall, Fig. 2 demonstrates that chatbot performance remains domain-dependent. ChatGPT-4/4o and Copilot/Bing generally showed the most favorable evidence landscape regarding accuracy and reliability, whereas readability outcomes were more variable across platforms. Importantly, no evaluated model consistently achieved specialist-level performance across all domains.

Based on these patterns, chatbot use was stratified into proposed levels of clinical supervision. Routine educational content was considered suitable only as adjunctive support, whereas evidence-sensitive claims, individualized risk framing, and treatment-planning recommendations required progressively greater professional verification or specialist-only decision-making (Table 3 and Fig. 3).

### 3.10 Certainty of the evidence

The certainty of evidence was judged very low across all prespecified outcomes using the GRADE framework^18^ (Supplementary Material 2).

Important methodological limitations persisted across the evidence base. Downgrading primarily resulted from risk of bias, imprecision, and methodological heterogeneity involving comparator definitions, evaluator calibration, prompting procedures, scoring frameworks, and outcome interpretation.

Additional concerns included small sample sizes, limited reproducibility assessments, the absence of standardized reference criteria, and the inherently dynamic nature of evolving large language model platforms. Consequently, apparent similarities between chatbot and specialist performance should be interpreted cautiously and regarded as exploratory rather than confirmatory findings.

## 4 Discussion

The present systematic review critically re-examined the rapidly expanding literature on artificial intelligence-based conversational agents in orthodontics and identified a field characterized less by technological consensus than by methodological fragmentation. Although most included studies reported that chatbots generated coherent and superficially plausible orthodontic responses, the collective evidence does not currently support equivalence between conversational AI systems and specialist-provided orthodontic guidance.

A central finding emerging from this synthesis is that chatbot fluency frequently masks informational instability. Across studies, large language models often produced responses perceived as readable, accessible, and clinically acceptable during initial evaluation, yet specialist assessment repeatedly identified omissions, oversimplifications, insufficient contextualization, or incomplete risk framing 6, 7, 8, 9^,^^12^, ^13^, ^14^, ^15^, ^16^. This distinction is particularly relevant in orthodontics because incorrect clinical communication does not necessarily manifest as explicit factual inaccuracy. Rather, harm may arise through absence of nuance, inadequate qualification of indications, or failure to recognize patient-specific variability.

This issue became particularly evident in studies evaluating clear aligners, orthognathic surgery, interceptive orthodontics, and treatment-planning scenarios. Abu Arqub et al.^12^ demonstrated that a substantial proportion of ChatGPT-generated clear-aligner claims were either incomplete or objectively false, including statements involving airway effects, treatment stability, and surgical implications. Similarly, treatment-planning studies showed that chatbot-generated recommendations frequently diverged from orthodontist-derived therapeutic reasoning when confronted with biomechanically or diagnostically complex cases.^24^ These findings reinforce that conversational coherence should not be confused with clinical validity.

Importantly, the present review identified that the main source of uncertainty was not merely chatbot performance itself, but rather the profound heterogeneity underlying the evidence base. Studies differed substantially regarding prompt engineering, session resetting, evaluator calibration, comparator definitions, outcome thresholds, and scoring systems 5, 6, 7, 8, 9^,^^13^, ^14^, ^15^, ^16^. In several investigations, minor changes in prompt wording or interaction structure produced measurable differences in chatbot outputs, indicating that these systems function as interaction-dependent probabilistic generators rather than stable knowledge repositories. Consequently, reported “accuracy” represents a contextual phenomenon strongly influenced by methodological design rather than an intrinsic property of the model alone.

This heterogeneity has important implications for the interpretation of “agreement with specialists.” Reviewer concerns regarding variability in orthodontic philosophies are highly relevant within this context. Orthodontics does not operate under universally identical therapeutic paradigms; treatment decisions may legitimately differ according to biomechanical philosophy, esthetic priorities, extraction preference, airway considerations, retention philosophy, and clinician experience. Therefore, disagreement between chatbot outputs and specialist recommendations should not always be interpreted as binary evidence of failure. Conversely, apparent agreement should not automatically be interpreted as proof of clinical correctness. Rather, agreement outcomes reflect concordance with the specific evaluative framework adopted within each study ^8^^,^^11^^,^^24^^,^^27^^,^^28^.

This distinction also explains why comparator definitions emerged as a major methodological limitation across the included literature. Some investigations compared chatbot responses against individual orthodontists, whereas others relied on consensus panels, institutional educational material, textbooks, predefined answer keys, or evidence-based summaries.^6^^,^^8^^,^^11^^,^^14^^,^^15^^,^^24^ These reference standards are not conceptually interchangeable. A chatbot response judged “adequate” under one framework could be considered incomplete or inappropriate under another. The absence of standardized orthodontic reference frameworks therefore substantially limits direct comparability across studies.

A key distinction must be made between diagnostic AI and conversational AI in orthodontics. Although recent evidence suggests promising performance of AI in image-based diagnostic tasks, such as automated detection of orthodontic root resorption, diagnostic and conversational systems operate under fundamentally different paradigms 1, 2, 3, 4. Diagnostic models are typically designed for narrow, task-specific pattern recognition, whereas large language models generate probabilistic responses across broader clinical contexts. Therefore, performance in radiographic detection should not be extrapolated to patient-facing conversational reliability, particularly because chatbots may influence patient expectations, treatment perceptions, and healthcare decision-making 1, 2, 3^,^^5^, ^6^, ^7^, ^8^, ^9^^,^^27^^,^^29^.

### 4.1 Comparison with previous evidence syntheses

This systematic review differs from the published synthesis by Santhosh Kumar et al.,^17^ in both scope and analytic emphasis. Whereas the review published in the Journal of the American Dental Association (JADA) primarily examined patient trust in AI-generated orthodontic advice, the present review synthesized a broader set of performance domains, including accuracy, completeness, readability, agreement with specialists, treatment-planning concordance, risk of misleading information, comparator heterogeneity, and certainty of evidence. It also incorporated additional recent studies and newer chatbot architectures, including ChatGPT-4o, Gemini 1.5 Pro, Claude 3.5 Sonnet, OpenEvidence, and MediSearch. Accordingly, the present review should be interpreted as a broader methodological and clinical synthesis rather than a duplicate review focused only on trust-related outcomes 5, 6, 7, 8, 9, 10, 11, 12, 13, 14, 15, 16^,^^24^^,^^25^.

At the same time, the findings should not be interpreted in an alarmist manner. Several studies consistently demonstrated that chatbots may serve useful supportive roles for reinforcement of routine information, clarification of basic orthodontic concepts, and improvement of informational accessibility.^7^^,^^8^^,^^10^^,^^25^^,^^30^ Their capacity for continuous availability, multilingual communication, and rapid content generation may become particularly valuable in settings with reduced specialist access or high informational demand. However, the evidence synthesized here strongly suggests that such systems should currently be regarded as adjunctive educational tools rather than substitutes for individualized orthodontic evaluation.

This distinction becomes even more relevant when considering ethical and regulatory implications. Large language models are fundamentally designed to generate statistically plausible language sequences, not to assume professional accountability or perform genuine clinical reasoning.^1^^,^^2^^,^^29^^,^^31^^,^^32^ As conversational AI becomes increasingly integrated into healthcare environments, the boundary between informational support and clinical advice may become progressively blurred ^1^^,^^2^^,^^29^, ^30^, ^31^, ^32^. This raises important concerns involving informed consent, liability, transparency of limitations, disclosure of uncertainty, and patient autonomy. Current evidence does not yet establish sufficient reliability for unsupervised orthodontic guidance 12, 13, 14^,^^25^.

The review also highlights several priorities for future investigations. First, substantial methodological harmonization is urgently needed. Development of standardized orthodontic question banks, consensus-derived reference answers, validated scoring systems, and calibrated evaluator frameworks would substantially improve comparability across studies 5, 6, 7, 8, 9^,^^11^, ^12^, ^13^, ^14^, ^15^, ^16^^,^^24^^,^^25^^,^^33^. Second, future research should move beyond descriptive evaluations of chatbot responses and investigate real-world effects on patient understanding, treatment acceptance, anxiety, behavioral modification, and clinical outcomes.^30^ Third, longitudinal monitoring of model evolution is essential because chatbot architectures change rapidly over time, potentially altering performance profiles within short intervals.^13^^,^^14^^,^^25^ Finally, interdisciplinary collaboration between orthodontists, computer scientists, ethicists, behavioral scientists, and regulatory agencies will be essential to ensure that technological advancement proceeds alongside safeguards for patient welfare.^1^^,^^2^^,^^31^^,^^32^

From an evidence-synthesis perspective, the present review underscores an important conceptual issue frequently overlooked in discussions surrounding generative AI: technological sophistication does not automatically translate into evidentiary maturity 1, 2, 3^,^^5^. Current enthusiasm surrounding large language models may inadvertently encourage premature clinical extrapolation before adequate validation standards are established.^1^^,^^2^^,^^29^^,^^31^^,^^32^ By critically synthesizing the presently fragmented literature, this review aims to provide a more balanced framework for interpreting chatbot performance in orthodontics, one that recognizes both the substantial communicative potential of these systems and the significant methodological and clinical limitations that continue to constrain their safe implementation 5, 6, 7, 8, 9, 10, 11, 12, 13, 14, 15, 16^,^^25^.

### 4.2 Clinical implications

Artificial intelligence chatbots are increasingly used by patients seeking orthodontic information before professional consultation 5, 6, 7, 8, 9^,^^11^^,^^30^. Current evidence suggests that these systems may assist with the delivery of routine educational content and reinforcement of general orthodontic concepts.^7^^,^^8^^,^^25^^,^^30^^,^^33^ However, important limitations persist regarding individualized interpretation, treatment planning, contextual risk framing, and specialist-level clinical reasoning 12, 13, 14, 15, 16^,^^24^. Orthodontists should therefore recognize that chatbot-generated information may influence patient expectations, perceptions, and treatment decisions even before clinical examination occurs.^27^^,^^29^^,^^31^^,^^32^ The proposed clinical supervision framework summarizes these practical boundaries by distinguishing low-risk adjunctive educational uses from evidence-sensitive applications and specialist-only decisions. At present, conversational AI systems should be regarded as supportive informational tools requiring professional supervision rather than autonomous sources of orthodontic guidance.^1^^,^^2^

## 5 Conclusion

The main conclusions of this review are as follows:

1. Accuracy and reliability: AI-based chatbots generally produced coherent and clinically plausible answers to routine orthodontic questions, but accuracy was inconsistent across model versions, prompts, comparators, and clinical domains.
2. Readability and accessibility: Chatbot-generated responses were often readable and accessible, particularly for foundational concepts and patient frequently asked questions; however, readability did not ensure clinical adequacy.
3. Patient preference: Some studies indicated that patients may prefer AI-generated explanations because they appear comprehensive and easy to understand, but patient preference should not be interpreted as evidence of correctness or safety.
4. Treatment planning: Current evidence does not support autonomous chatbot use for diagnosis, individualized recommendations, treatment-planning decisions, or complex case-based orthodontic reasoning.
5. Clinical applicability: Conversational AI may be useful as an adjunctive educational tool under professional supervision, especially for routine information, but outputs require verification before clinical or patient-facing use.
6. Certainty of evidence: Because the certainty of evidence was very low, future studies should use standardized prompts, validated scoring tools, calibrated evaluators, transparent model reporting, and real-world outcome assessment.

## Patient consent statement

Patient consent was not required because this study did not involve human participants or identifiable patient data.

## Ethics approval statement

Ethical approval was not required because this study is a systematic review of previously published literature.

## Registration

This systematic review was prospectively registered in PROSPERO (CRD420261443844).

## Animal studies

This study did not involve animals.

## Permission to reproduce material

Not applicable.

## Data availability statement

All data extracted and analyzed in this systematic review are available within the article and its supplementary materials. No new datasets or analytic code were generated.

## Human and animal statements

This study did not involve any human/animal participants or patient data so this consent is not required.

## Funding sources

This research did not receive any specific grant from funding agencies in the public, commercial, or not-for-profit sectors.

## Declaration of competing interest

The authors declare that they have no known competing financial interests or personal relationships that could have appeared to influence the work reported in this paper.
