# Supplementary material for: Conversational artificial intelligence compared with orthodontic reference standards for orthodontic information: A systematic review of accuracy, readability, safety, and certainty of evidence
Source: J Oral Biol Craniofac Res. 2026 Sep 27;16(6):101552. doi: 10.1016/j.jobcr.2026.101552 (PMC13639733; doi:10.1016/j.jobcr.2026.101552)
Supplement: Supplementary file 1 [file mmc2.pdf]

Author(s):
Question: AI-based chatbots compared to Orthodontic specialists for patients seeking orthodontic information
Setting: Orthodontic information environment
Bibliography:

Certainty assessment							№ of patients		Effect		Certainty	Importance
№ of studies	Study design	Risk of bias	Inconsistency	Indirectness	Imprecision	Other considerations	AI-based chatbots	Orthodontic specialists	Relative (95% CI)	Absolute (95% CI)		
**Accuracy / Reliability (assessed with: Expert judgement, predefined scoring systems, correctness against reference answers)**												
16	non-randomised studies	serious[a]	not serious	not serious	serious[b]	none	0/0	0/0	not estimable		⊕◯◯◯ Very low[a,b]	CRITICAL
**Completeness of Information (assessed with: Coverage of key diagnostic, therapeutic or procedural elements)**												
12	non-randomised studies	serious[c]	not serious	not serious	serious[d]	none	0/0	0/0	not estimable		⊕◯◯◯ Very low[c,d]	CRITICAL
**Agreement with specialists (assessed with: Preference, ranking, concordance with orthodontist responses)**												
9	non-randomised studies	serious[e]	not serious	not serious	serious[f]	none	0/0	0/0	not estimable		⊕◯◯◯ Very low[e,f]	CRITICAL
**Readability / Accessibility**												
5	non-randomised studies	serious[g]	not serious	not serious	serious[h]	none	0/0	0/0	not estimable		⊕◯◯◯ Very low[g,h]	CRITICAL
**Risk of misleading or Harmful information**												
8	non-randomised studies	serious[i]	not serious	not serious	serious[j]	none	0/0	0/0	not estimable		⊕◯◯◯ Very low[i,j]	CRITICAL

CI: confidence interval

Explanations

a. Most included studies relied on subjective expert judgment, non-validated scoring systems, and lacked assessor blinding. Considerable methodological heterogeneity and absence of standardized reference criteria increase the likelihood of bias.
b. The available evidence is derived from relatively small samples, heterogeneous assessment methods, and narrative reporting without pooled quantitative estimates. The absence of precise effect measures limits confidence in the magnitude and stability of the findings.
c. Completeness assessments were primarily based on author-defined or expert-based criteria, frequently without validation or calibration procedures. Lack of standardized instruments and potential evaluator subjectivity may have influenced the estimates.
d. Estimates were based on limited datasets and descriptive approaches, with no quantitative synthesis or precision metrics. Consequently, the certainty regarding the true extent of information completeness remains limited.
e. Measures of agreement were often derived from preference or ranking approaches involving limited numbers of evaluators. Absence of blinding and potential authority bias may have affected outcome assessment.
f. Agreement outcomes relied on small numbers of evaluators and non-pooled results, frequently without measures of variability. This restricts the precision and reliability of the estimated effects.
g. Readability and accessibility assessments were based on different readability formulas, subjective interpretability judgments, and heterogeneous patient-information contexts. Several studies lacked standardized evaluation procedures.
h. Although readability metrics were reported in some studies, analyses were generally based on limited datasets and non-pooled evidence, reducing confidence in the precision and generalizability of the findings.
i. The assessment of potentially misleading or harmful information was highly subjective; definitions varied across studies, and most investigations lacked standardized harm-detection frameworks.
j. The identification of misleading or harmful content was commonly descriptive, based on small study samples and without quantitative aggregation, leading to uncertainty regarding the stability of the estimates.
