# Supplementary material for: Conversational artificial intelligence compared with orthodontic reference standards for orthodontic information: A systematic review of accuracy, readability, safety, and certainty of evidence
Source: J Oral Biol Craniofac Res. 2026 Sep 27;16(6):101552. doi: 10.1016/j.jobcr.2026.101552 (PMC13639733; doi:10.1016/j.jobcr.2026.101552)
Supplement: Supplementary file 2 [file mmc1.docx]

**Supplementary Table S1.** Electronic search strategies and the number of records retrieved from each database

| **Database** | **n** | **Search Strategy** |
| --- | --- | --- |
| Embase | 978 | ('orthodontics'/exp OR orthodontic*:ti,ab OR orthodontics:ti,ab OR 'orthodontic treatment':ti,ab OR 'clear aligner*':ti,ab OR braces:ti,ab OR retainer*:ti,ab OR 'orthognathic surgery':ti,ab) AND ('artificial intelligence'/exp OR 'machine learning'/exp OR chatbot*:ti,ab OR 'ai chatbot*':ti,ab OR 'large language model*':ti,ab OR llm*:ti,ab OR chatgpt:ti,ab OR gpt:ti,ab OR bard:ti,ab OR gemini:ti,ab OR copilot:ti,ab OR bing:ti,ab OR claude:ti,ab OR openevidence:ti,ab OR medisearch:ti,ab) AND (accuracy:ti,ab OR reliability:ti,ab OR readability:ti,ab OR completeness:ti,ab OR validity:ti,ab OR evaluation:ti,ab OR assessment:ti,ab OR agreement:ti,ab OR safety:ti,ab OR misleading:ti,ab OR harmful:ti,ab OR 'reference standard*':ti,ab OR expert*:ti,ab OR specialist*:ti,ab) AND ('cross-sectional':ti,ab OR comparative:ti,ab OR survey:ti,ab OR 'content analysis':ti,ab OR validation:ti,ab OR evaluative:ti,ab OR analytical:ti,ab OR observational:ti,ab) AND [2022-2026]/py |
| Web Of Science | 589 | TS=(orthodontic* OR orthodontics OR 'orthodontic treatment' OR 'clear aligner*' OR braces OR retainer* OR 'orthognathic surgery') AND TS=('artificial intelligence' OR AI OR 'machine learning' OR chatbot* OR 'AI chatbot*' OR 'large language model*' OR LLM* OR ChatGPT OR GPT OR Bard OR Gemini OR Copilot OR Bing OR Claude OR OpenEvidence OR MediSearch) AND TS=(accuracy OR reliability OR readability OR completeness OR validity OR evaluation OR assessment OR agreement OR safety OR misleading OR harmful OR 'reference standard*' OR expert* OR specialist*) AND TS=('cross-sectional' OR comparative OR survey OR 'content analysis' OR validation OR evaluative OR analytical OR observational) |
| Pubmed | 888 | (orthodontic*[Title/Abstract] OR orthodontics[MeSH Terms] OR 'orthodontic treatment'[Title/Abstract] OR 'clear aligner*'[Title/Abstract] OR braces[Title/Abstract] OR retainer*[Title/Abstract] OR 'orthognathic surgery'[Title/Abstract]) AND ('Artificial Intelligence'[Mesh] OR 'Machine Learning'[Mesh] OR chatbot*[Title/Abstract] OR 'AI chatbot*'[Title/Abstract] OR 'large language model*'[Title/Abstract] OR LLM*[Title/Abstract] OR ChatGPT[Title/Abstract] OR GPT[Title/Abstract] OR Bard[Title/Abstract] OR Gemini[Title/Abstract] OR Copilot[Title/Abstract] OR Bing[Title/Abstract] OR Claude[Title/Abstract] OR OpenEvidence[Title/Abstract] OR MediSearch[Title/Abstract]) AND (accuracy[Title/Abstract] OR reliability[Title/Abstract] OR readability[Title/Abstract] OR completeness[Title/Abstract] OR validity[Title/Abstract] OR evaluation[Title/Abstract] OR assessment[Title/Abstract] OR agreement[Title/Abstract] OR safety[Title/Abstract] OR misleading[Title/Abstract] OR harmful[Title/Abstract] OR 'reference standard*'[Title/Abstract] OR expert*[Title/Abstract] OR specialist*[Title/Abstract]) AND ('cross-sectional'[Title/Abstract] OR comparative[Title/Abstract] OR survey[Title/Abstract] OR 'content analysis'[Title/Abstract] OR validation[Title/Abstract] OR evaluative[Title/Abstract] OR analytical[Title/Abstract] OR observational[Title/Abstract]) AND ('2022/01/01'[Date - Publication] : '3000'[Date - Publication]) |
| Scopus | 160 | TITLE-ABS-KEY(orthodontic* OR orthodontics OR 'orthodontic treatment' OR 'clear aligner*' OR braces OR retainer* OR 'orthognathic surgery') AND TITLE-ABS-KEY(ChatGPT OR chatbot* OR 'large language model*' OR LLM* OR Gemini OR Bard OR Copilot OR Bing OR Claude OR OpenEvidence OR MediSearch) AND TITLE-ABS-KEY(accuracy OR reliability OR readability OR completeness OR validity OR evaluation OR assessment OR agreement OR safety OR misleading OR harmful OR 'reference standard*' OR expert* OR specialist*) AND TITLE-ABS-KEY('cross-sectional' OR comparative OR survey OR 'content analysis' OR validation OR evaluative OR analytical OR observational) |
| Open Science Framework | 0 | ('chatbot' OR 'AI chatbot' OR 'large language model' OR LLM OR ChatGPT OR Bard OR Gemini OR Copilot) AND (orthodontic OR orthodontics) AND (evaluation OR accuracy OR reliability OR readability OR agreement OR validation OR survey OR content analysis OR comparative) |
| ClinicalTrials.gov | 9 | (chatbot OR "artificial intelligence" OR "large language model" OR ChatGPT OR GPT OR Bard OR Gemini OR Copilot) AND (orthodontic OR orthodontics OR "clear aligner" OR braces OR retainer OR "orthognathic surgery") |
| Google Scholar | 70 | Combined searches using: ChatGPT orthodontics patient questions accuracy reliability; AI chatbot orthodontics patient information readability; large language model orthodontics evaluation; ChatGPT clear aligners orthodontics content analysis; ChatGPT orthognathic surgery patient information quality; Gemini Copilot Bard ChatGPT orthodontics comparative study. |

*Searches were conducted using combinations of controlled vocabulary and free-text terms related to conversational artificial intelligence and orthodontics. Search syntax was adapted according to the indexing structure and retrieval rules of each database while maintaining conceptual equivalence across platforms. Filters related to publication years and subject areas were applied when supported. The number of retrieved records (n) reflects the results identified at the time of the final search.* *The final search was conducted on May 9, 2026.*

**Supplementary Table S2.** Artificial intelligence chatbot systems evaluated in the included studies, including developer information, model version, access date, and availability

| **Study** | **AI models tested** | **Developer** | **Version/access date** | **Access type** |
| --- | --- | --- | --- | --- |
| Abu Arqub et al., 2024 | ChatGPT | OpenAI | Version not reported; responses collected from chat.openai.com. | NR |
| Alkhamees, 2024 | ChatGPT-4 | OpenAI | ChatGPT version 4; access date NR. | NR |
| Arslan et al., 2024 | ChatGPT (GPT-3.5); Google Bard | OpenAI; Google | ChatGPT GPT-3.5 free version as of March 2023; Google Bard NR. | ChatGPT free; Bard free |
| Daraqel et al., 2024 | ChatGPT-3.5; Google Bard | OpenAI; Google | ChatGPT-3.5; Google Bard accessed July 16, 2023. | Both free |
| Dursun and Bilici Gecer, 2024 | ChatGPT-3.5; ChatGPT-4; Gemini; Copilot | OpenAI; Google; Microsoft | GPT-3.5; GPT-4; Gemini described as LaMDA/PaLM 2-based and relaunched as Gemini; Copilot relaunched from Bing Chat and uses GPT-4 technology. | ChatGPT-3.5 free; ChatGPT-4 paid; Gemini and Copilot publicly available |
| Hatia et al., 2024 | ChatGPT-4 | OpenAI | ChatGPT version 4; questions entered on April 21, 2023. | NR |
| Kilinc and Mansiz, 2024 | ChatGPT March 23, 2023 version; ChatGPT May 24, 2023 version | OpenAI | First evaluated April 20, 2023; second evaluated July 12, 2023. | Unclear |
| Kurt Demirsoy et al., 2024 | ChatGPT-4.0 | OpenAI | ChatGPT version 4.0; access date NR. | NR |
| Makrygiannakis et al., 2024 | ChatGPT-3.5; ChatGPT-4; Google Bard; Microsoft Bing Chat | OpenAI; Google; Microsoft | ChatGPT-3.5; ChatGPT-4; Google Bard LaMDA/PaLM 2; Microsoft Bing Chat GPT-4-based. | ChatGPT-3.5 free; ChatGPT-4 paid; Bard/Bing NR |
| Metin and Goymen, 2025 | ChatGPT-4; ChatGPT-4o; Microsoft Copilot; Google Gemini 1.5 Pro; Claude 3.5 Sonnet | OpenAI; Microsoft; Google; Anthropic | Accessed December 2024. | NR |
| Naureen and Kiani, 2024 | ChatGPT-4; Google Bard | OpenAI; Google | Study conducted June 23 to August 30, 2023; exact access dates NR. | NR |
| Salmanpour et al., 2025 | ChatGPT-4.0; Microsoft Copilot | OpenAI; Microsoft | Questions posed between October 5 and October 25, 2024; Copilot powered by GPT-4 as described by authors. | ChatGPT-4.0 paid; Copilot free |
| Tanaka et al., 2025 | ChatGPT-3.5; Microsoft Bing Copilot; Google Bard/Gemini | OpenAI; Microsoft; Google | All accessed February 2, 2024. | Free versions reported |

*(Supplementary Table S2. Continuation)*

| **Study** | **AI models tested** | **Developer** | **Version/access date** | **Access type** |
| --- | --- | --- | --- | --- |
| Vassis et al., 2024 | ChatGPT-3.5; ChatGPT-4.0 | OpenAI | October 2023 version of ChatGPT; GPT-3.5 and GPT-4.0 used. | ChatGPT-3.5 free; GPT-4.0 paid |
| Yurdakurban et al., 2024 | ChatGPT-4; OpenEvidence; MediSearch | OpenAI; OpenEvidence; MediSearch | Version/access date NR. | NR |
| Zhou et al., 2025 | ChatGPT-4 | OpenAI | June 13, 2023 version; accessed September 16, 2023. | NR |

*Information regarding AI chatbot systems, developer companies, model versions, access dates, and availability type was extracted as reported in the original studies. Because large language models evolve continuously, the characteristics summarized in this table correspond to the versions available at the time each investigation was conducted and may not reflect current platform configurations. NR = not reported.*

**Supplementary Table S3.** Characteristics of the datasets, origin of the questions, thematic coverage, language, and prompt-engineering strategies adopted in the included studies.

| **Study** | **Total questions/cases** | **Source of questions/cases** | **Question type** | **Orthodontic topics** | **Language** | **Prompt methodology** |
| --- | --- | --- | --- | --- | --- | --- |
| Abu Arqub et al., 2024 | 111 | Generated by orthodontists using predefined clear-aligner outcome domains and subdomains; refined by discussion. | Open-ended CAT queries | Clear aligner treatment outcomes, claims, limitations, stability, airway, root control, pain, effectiveness and related patient/clinician concerns. | English | One author collected ChatGPT answers from chat.openai.com; session-reset procedures not reported; answers scored independently by five CAT experts after calibration/pilot scoring. |
| Alkhamees, 2024 | 60 | ChatGPT-4 generated 20 frequently asked questions for each of three orthodontic topics and then answered them. | Patient-oriented FAQs | Impacted canines; interceptive orthodontic treatment; orthognathic surgery. | English | ChatGPT-4 generated and answered questions; all answers were saved and independently rated by five orthodontists using predefined criteria. |
| Arslan et al., 2024 | 24 | Frequently asked questions gathered from the first 50 pages of Google search results. | Open-ended FAQs | Conventional braces, clear aligners, orthognathic surgery, orthodontic retainers. | English | Past searches were cleared before identifying Google FAQs. Whether a new AI session was used for each prompt was not explicitly stated. |
| Daraqel et al., 2024 | 100 | Consensus question bank developed by a panel of five orthodontic specialists. | Open-ended FAQs | Age-specific treatment, clear aligners, combined orthodontic-orthognathic treatment, digital orthodontics, expansion, extraction, impacted teeth, mini-implants, treatment information and retention. | English | A single investigator submitted each question once to both models; chat window was refreshed for every question to minimize carryover. |
| Dursun and Bilici Gecer, 2024 | 20 | FAQs from the first 30 non-sponsored websites found through Google search for clear-aligner FAQs. | Open-ended FAQs | Clear aligner function, comparison with braces, indications, benefits, effectiveness, cost, insurance, wear/care, treatment process, side effects, attachments and IPR. | English | Each question was posed once in a separate chat window for each model. The first Gemini draft and Copilot balanced mode were used. |

*(Supplementary Table S3. Continuation)*

| **Study** | **Total questions/cases** | **Source of questions/cases** | **Question type** | **Orthodontic topics** | **Language** | **Prompt methodology** |
| --- | --- | --- | --- | --- | --- | --- |
| Hatia et al., 2024 | 21 open-ended questions and 7 clinical scenarios | Ten specialized orthodontists from ten Italian postgraduate schools developed questions and scenarios based on interceptive orthodontics subspecialties and real clinical contexts. | Open-ended clinical questions and clinical scenarios | Class II and III malocclusion, cephalometric analysis, dental inclusion, open bite, atypical swallowing, deep bite, interceptive diagnosis and management. | English | A single researcher entered all prompts into ChatGPT-4 on April 21, 2023. The model was instructed to consider available guidelines; for clinical scenarios it was asked to identify the most probable diagnosis. |
| Kilinc and Mansiz, 2024 | 34 | FAQs about orthodontics collected from the first 60 websites in Google Search; 307 questions from 41 eligible websites were screened and reduced to 34. | Open-ended FAQs | General orthodontic questions and treatment-related questions about braces, orthodontists, duration and appliances. | English | The same 34 questions were posed to two different ChatGPT versions on different dates; detailed session-control methods were not fully reported. |
| Kurt Demirsoy et al., 2024 | 4 | Authors prepared frequently asked/curious questions across four core orthodontic areas. | Broad patient/education questions | Clear aligners, lingual orthodontics, esthetic braces, temporomandibular disorders. | English | Four questions were asked to ChatGPT-4.0 and the same responses were evaluated by orthodontists, final-year dental students and individuals seeking orthodontic treatment. |
| Makrygiannakis et al., 2024 | 10 | Questions agreed by authors and benchmarked against evidence from consensus statements, systematic reviews and PubMed. | Open-ended clinical questions | Common clinically relevant orthodontic issues. | NR | Each question was posed once to each LLM in a new session, without follow-up questions or rephrasing. |
| Metin and Goymen, 2025 | 40 | Topics derived from 100 Google websites; 155 textbook-based true/false items reduced to 40. | True/false questions | Growth, dentition, biomechanics, diagnosis, malocclusion, appliances, treatment planning, retention and relapse. | English | Three sessions on consecutive days with cleared history/cookies and different computers. |

*(Supplementary Table S3. Continuation)*

| **Study** | **Total questions/cases** | **Source of questions/cases** | **Question type** | **Orthodontic topics** | **Language** | **Prompt methodology** |
| --- | --- | --- | --- | --- | --- | --- |
| Naureen and Kiani, 2024 | 90 | Thirty questions per category derived from recent review articles; standard answer key used for comparison. | Knowledge-based questions | Mini-implant-assisted rapid palatal expansion, clear aligners, cone-beam computed tomography in orthodontics. | English | Questions were presented to ChatGPT-4 and Google Bard; two independent raters scored responses against a standard key after pilot reliability assessment. |
| Salmanpour et al., 2025 | 15 | Randomly selected from AAO FAQs after senior clinicians screened commonly asked items. | Open-ended patient FAQs | Pain management, appliance care, treatment duration, hygiene, diet and post-treatment guidance. | English | Each question was presented once to each chatbot; chat window was refreshed before each question and no prompt optimization was applied. |
| Tanaka et al., 2025 | 1 clinical case | A single clinical case selected for typical orthodontic challenges and benchmarked against an experienced orthodontist plan. | Open-ended clinical case/treatment planning | Mixed dentition interceptive treatment with impacted maxillary central incisor, anterior crossbite and constricted maxilla. | NR | A detailed clinical case description was presented to each AI platform in a new session; AI-generated and orthodontist treatment plans were evaluated by orthodontists. |
| Vassis et al., 2024 | 28 self-formulated prompts and standardized outputs; 46-patient evaluation. | Patients generated prompts about orthodontic side effects; a standardized prompt was also used; AI text was compared with institutional standard patient information. | Self-formulated and standardized patient-education prompts | Pretreatment education and side effects of orthodontic treatment. | English | Standardized prompt on orthodontic treatment side effects; expert and patient evaluation. |
| Yurdakurban et al., 2024 | 60 | Guidelines on orthognathic surgery were reviewed and patient questions were produced by two researchers. | Open-ended patient questions | Orthognathic surgery: general information/procedure and results/recovery. | English | Sixty questions were submitted to ChatGPT-4, OpenEvidence and MediSearch; responses were evaluated for quality, reliability, readability and similarity using structured tools. |

*(Supplementary Table S3. Continuation)*

| **Study** | **Total questions/cases** | **Source of questions/cases** | **Question type** | **Orthodontic topics** | **Language** | **Prompt methodology** |
| --- | --- | --- | --- | --- | --- | --- |
| Zhou et al., 2025 | 30 | Selected from the Chinese book Medical Guidance for Dental and Maxillofacial Deformities. | Open-ended FAQs | Pre-treatment first visit, during-treatment plan meeting and post-treatment questions. | Chinese | Each question was entered as a separate independent prompt using the New Chat feature. |

*Dataset composition, prompt wording, thematic coverage, and interaction procedures were extracted directly from the included studies. Considerable variability existed regarding question origin, prompt standardization, session resetting, history deletion, and use of standardized versus self-formulated prompts. Such methodological heterogeneity limits direct comparability across studies and may influence chatbot performance outcomes.*

**Supplementary Table S4.** Summary of comparative performance outcomes and principal conclusions reported in the included studies.

| **Study** | **Accuracy results** | **Quality/reliability results** | **Readability** | **Key statistical findings** | **Best performing model/source** | **Main conclusion** |
| --- | --- | --- | --- | --- | --- | --- |
| Abu Arqub et al., 2024 | Mean accuracy score 2.6 +/- 1.1; 58% objectively true, 18% selected facts, 9% minimal facts and 15% false. | Suboptimal information quality with omitted facts and false claims in several CAT domains. | Not reported | Descriptive results identified false claims related to surgery need, airway function, root parallelism, sleep apnea and stability. | Single model: ChatGPT. | ChatGPT responses on clear aligners were frequently incomplete and sometimes false; clinician/patient caution is required. |
| Alkhamees, 2024 | Overall 68.7% of responses were rated good or very good across topics. | Generally good information but variable and not reliable enough to replace professional opinion. | Not reported | No significant difference between the three topics; low inter-rater agreement. | Single model: ChatGPT-4. | ChatGPT-4 can provide generally good patient information on selected orthodontic topics, but outputs require caution and professional verification. |
| Arslan et al., 2024 | ChatGPT slightly higher than Bard. | Similar trend favoring ChatGPT. | Not reported | Significant difference in numerical rating scales for both investigators. | ChatGPT. | Both systems were acceptable for common questions, with ChatGPT marginally superior. |
| Daraqel et al., 2024 | High accuracy for both; ChatGPT median 9 vs Bard median 8. | Completeness comparable; both median approximately 8. | Not reported | Accuracy significantly higher for ChatGPT, but magnitude small; Bard faster. | ChatGPT for accuracy; Bard for response time. | Both models produced accurate and complete responses to general orthodontic questions, with limited clinically meaningful differences. |

*(Supplementary Table S4. Continuation)*

| **Study** | **Accuracy results** | **Quality/reliability results** | **Readability** | **Key statistical findings** | **Best performing model/source** | **Main conclusion** |
| --- | --- | --- | --- | --- | --- | --- |
| Dursun and Bilici Gecer, 2024 | No significant difference in accuracy; ChatGPT-4 highest mean Likert score. | Copilot highest reliability and quality scores. | Generally difficult; Gemini most readable. | Several pairwise differences for reliability, quality and readability. | ChatGPT-4 for accuracy; Copilot for quality/reliability; Gemini for readability. | Chatbots may assist patient information on clear aligners but require evidence-based supplementation and improved readability. |
| Hatia et al., 2024 | Open questions and clinical scenarios had median accuracy 4.9/6; entirely correct responses in 40.5% and 46%, respectively. | Completeness median 2.4/3 for open questions and 2.5/3 for clinical cases; entirely complete responses in 50.5% and 54.3%. | Not reported | Descriptive and normality analyses; performance high but not perfect. | Single model: ChatGPT-4. | ChatGPT-4 showed high accuracy/completeness in interceptive orthodontics and clinical scenarios but cannot replace human clinical reasoning. |
| Kilinc and Mansiz, 2024 | Moderate reliability. | Reliability improved in later ChatGPT version. | Difficult; reading level often high school/college level. | Several significant changes between versions. | Updated ChatGPT version. | Reliability increased over time but remained moderate, and readability was not ideal for lay users. |
| Kurt Demirsoy et al., 2024 | Orthodontists tended to rate responses more critically than patients. | DISCERN and GQS indicated reasonable/medium-to-sound quality; patient scores often highest. | Not reported | Significant differences among orthodontists, students and patients for several topics. | Single model: ChatGPT-4.0. | ChatGPT has potential for patient information and education, but perceptions vary substantially by evaluator background. |

*(Supplementary Table S4. Continuation)*

| **Study** | **Accuracy results** | **Quality/reliability results** | **Readability** | **Key statistical findings** | **Best performing model/source** | **Main conclusion** |
| --- | --- | --- | --- | --- | --- | --- |
| Makrygiannakis et al., 2024 | Bing highest average score, followed by ChatGPT-4, Bard and ChatGPT-3.5. | Bing highest on comprehensiveness, scientific accuracy, clarity and relevance. | Not reported | Bing outperformed ChatGPT-3.5 and Bard; ChatGPT-4 outperformed ChatGPT-3.5. | Microsoft Bing Chat. | LLMs have evidence-support potential but may produce incomplete or inaccurate clinical answers; orthodontist judgment remains essential. |
| Metin and Goymen, 2025 | Orthodontists highest; AI chatbots generally above students and dentists; ChatGPT-4o closest to orthodontists. | Consistency varied; ChatGPT-4 most consistent, ChatGPT-4o and Claude least consistent. | Not reported | Multiple significant contrasts among clinician and chatbot groups. | ChatGPT-4o for accuracy; ChatGPT-4 for consistency. | AI chatbots may support education/guidance, but inconsistent responses create misinformation risk. |
| Naureen and Kiani, 2024 | ChatGPT-4 total score 92.6% vs Google Bard 72%. | Not assessed separately. | Not reported | Significant differences for MARPE and CBCT (p=0.001) and clear aligners (p=0.013). | ChatGPT-4. | ChatGPT-4 was more accurate than Google Bard for recent orthodontic treatment modalities. |
| Salmanpour et al., 2025 | Expert responses generally rated highest by patients and orthodontists. | ChatGPT-4.0 and Copilot acceptable but lower than experts in several critical items. | Reported; evaluated by Flesch-Kincaid. | Experts significantly outperformed chatbots in several questions; some items showed comparable chatbot performance. | Expert responses; among chatbots ChatGPT-4.0 generally stronger than Copilot. | Chatbots are acceptable adjuncts but do not match expert response adequacy. |

*(Supplementary Table S4. Continuation)*

| **Study** | **Accuracy results** | **Quality/reliability results** | **Readability** | **Key statistical findings** | **Best performing model/source** | **Main conclusion** |
| --- | --- | --- | --- | --- | --- | --- |
| Tanaka et al., 2025 | Orthodontist treatment plan received significantly higher agreement than AI-generated plans. | Orthodontist plan superior. | Not reported | p < 0.001 for orthodontist vs AI plans; no significant differences among AI platforms. | Experienced orthodontist. | Clinical judgment and experience remain essential for orthodontic treatment planning. |
| Vassis et al., 2024 | GPT-4 generally scored higher than GPT-3.5, but expert ratings were not thoroughly satisfactory. | AI-generated information often perceived by patients as useful/comprehensive; experts judged content limited. | Not formally assessed | Nearly 80% of patients preferred AI-generated text; expert ratings indicated only neither deficient nor satisfactory quality. | GPT-4 for expert validity; AI text for patient preference. | Patients may prefer AI-generated education, but content validity remains insufficient for standalone pretreatment education. |
| Yurdakurban et al., 2024 | Accuracy not the primary endpoint; data quality was generally high across chatbots. | MediSearch highest for reliability/GQS; OpenEvidence highest for EQIP and similarity-related metrics. | Difficult readability; college-level education required by SMOG. | Differences varied by instrument and chatbot. | Outcome-dependent: MediSearch for reliability/quality; OpenEvidence for EQIP/SMOG/similarity. | Orthognathic-surgery chatbot information was generally high quality/reliable but difficult to read and should not replace professional consultation. |
| Zhou et al., 2025 | ChatGPT-4 ranked first in 50.8% of rankings and had a better mean rank than both orthodontist groups. | Highest ranking for standardized FAQs. | Not reported | ChatGPT significantly outperformed Ortho A and Ortho B; performance correlated with inter-rater agreement. | ChatGPT-4. | Conversational AI may outperform orthodontists for standardized FAQs, especially when answers are broadly accepted among professionals. |

*(Supplementary Table S4. Continuation)*

*Descriptors such as higher, moderate, acceptable, comparable, significant, or superior reflect the terminology and interpretative thresholds adopted by the original authors and should not be interpreted as standardized effect measures. The best-performing model/source was study-specific and could vary according to the evaluated endpoint, including accuracy, reliability, readability, treatment-planning performance, agreement, or patient preference. Differences in study design, evaluator background, prompt methodology, and outcome assessment contributed substantially to heterogeneity across investigations.*
